# Performance of an ultra-fast deep-learning accelerated MRI screening protocol for prostate cancer compared to a standard multiparametric protocol

**DOI:** 10.1007/s00330-024-10776-7

**Published:** 2024-05-23

**Authors:** B. Oerther, H. Engel, A. Nedelcu, R. Strecker, T. Benkert, D. Nickel, E. Weiland, T. Mayrhofer, F. Bamberg, M. Benndorf, J. Weiß, C. Wilpert

**Affiliations:** 1 Department of Radiology, Medical Center – University of Freiburg, Faculty of Medicine, University of Freiburg, Freiburg im Breisgau, Germany; 2 MR Application Predevelopment, Siemens Healthineers GmbH, Erlangen, Germany; 3 EMEA Scientific Partnerships, Siemens Healthineers GmbH, Erlangen, Germany; 4 School of Business Studies, Stralsund University of Applied Sciences, Stralsund, Germany; 5 Cardiovascular Imaging Research Center, Department of Radiology, Massachusetts General Hospital and Harvard Medical School, Boston, MA USA

**Keywords:** Prostate cancer screening, Deep learning, MRI efficiency, Biparametric protocol, Multiparametric MRI

## Abstract

**Objectives:**

To establish and evaluate an ultra-fast MRI screening protocol for prostate cancer (PCa) in comparison to the standard multiparametric (mp) protocol, reducing scan time and maintaining adequate diagnostic performance.

**Materials and methods:**

This prospective single-center study included consecutive biopsy-naïve patients with suspected PCa between December 2022 and March 2023. A PI-RADSv2.1 conform mpMRI protocol was acquired in a 3 T scanner (scan time: 25 min 45 sec). In addition, two deep-learning (DL) accelerated sequences (T2- and diffusion-weighted) were acquired, serving as a screening protocol (scan time: 3 min 28 sec). Two readers evaluated image quality and the probability of PCa regarding PI-RADSv2.1 scores in two sessions. The diagnostic performance of the screening protocol with mpMRI serving as the reference standard was derived. Inter- and intra-reader agreements were evaluated using weighted kappa statistics.

**Results:**

We included 77 patients with 97 lesions (mean age: 66 years; SD: 7.7). Diagnostic performance of the screening protocol was excellent with a sensitivity and specificity of 100%/100% and 89%/98% (cut-off ≥ PI-RADS 4) for reader 1 (R1) and reader 2 (R2), respectively. Mean image quality was 3.96 (R1) and 4.35 (R2) for the standard protocol vs. 4.74 and 4.57 for the screening protocol (*p* < 0.05). Inter-reader agreement was moderate (*κ*: 0.55) for the screening protocol and substantial (*κ*: 0.61) for the multiparametric protocol.

**Conclusion:**

The ultra-fast screening protocol showed similar diagnostic performance and better imaging quality compared to the mpMRI in under 15% of scan time, improving efficacy and enabling the implementation of screening protocols in clinical routine.

**Clinical relevance statement:**

The ultra-fast protocol enables examinations without contrast administration, drastically reducing scan time to 3.5 min with similar diagnostic performance and better imaging quality. This facilitates patient-friendly, efficient examinations and addresses the conflict of increasing demand for examinations at currently exhausted capacities.

**Key Points:**

- *Time-consuming MRI protocols are in conflict with an expected increase in examinations required for prostate cancer screening.*
- *An ultra-fast MRI protocol shows similar performance and better image quality compared to the standard protocol.*
- *Deep-learning acceleration facilitates efficient and patient-friendly examinations, thus improving prostate cancer screening capacity.*

**Graphical Abstract:**

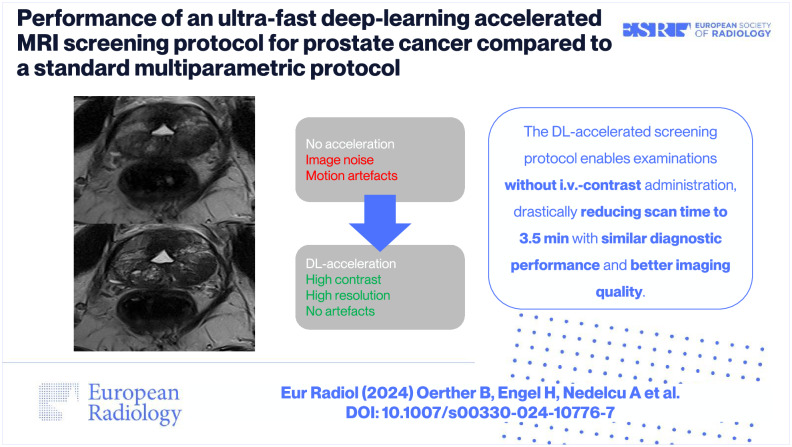

## Introduction

MpMRI serves as the fundamental imaging tool in the diagnostic pathway of PCa and has subsequently been implemented in multiple internationally recognized guidelines [1, 2]. It consists of a multitude of sequences, which enable the reader to rate the examination regarding the likelihood of PCa according to a standardized reporting system (Prostate Imaging Reporting and Data System; PI-RADS), currently in version 2.1 [3]. Categorization then determines further clinical management: PI-RADS scores of > 3 usually entail prostate biopsy, PI-RADS 3 lesions may receive biopsy depending on PSA density (PSAD) [2]. Qualitative imaging features translate into scores according to an evidence-based algorithm, mainly relying on T2-weighted (T2w) sequences and diffusion-weighted imaging (DWI). Whereas high-resolution T2w sequences provide excellent soft tissue contrast and facilitate morphological evaluation, DWI allows functional assessment. T1-weighted sequences offer additional anatomical information and intraprostatic hemorrhage detection. Dynamic contrast-enhanced (DCE) sequences have lost importance as they only impact the score in the case of a PI-RADS 3 lesion of the peripheral zone [3]. Their benefit has further been questioned in a recent prospective study that found a significant decrease of csPCa in DCE-positive PI-RADS 3 lesions compared to PI-RADS 4 lesions, recommending biopsy for PI-RADS 3 and DCE-positive PI-RADS 3 lesions alike only in case of an increased PSAD [4]. Therefore, the implementation of DCE imaging in future iterations of PI-RADS seems debatable.

Extensive multiparametric protocols require a surplus of scan time compared to abbreviated protocols, therefore limiting the capacity for examinations per day. Hence, biparametric (bp) protocols have gained in interest, omitting the DCE sequences and contrast medium (CM)-related adversities. These protocols can be abbreviated even further by reducing T2w sequences to a single plane [5]. Both bp and abbreviated bp protocols have shown similar diagnostic accuracy in the detection of csPCa [6, 7]. Although significantly faster than mpMRI, bp protocols are still time-consuming, mainly due to the long acquisition times of T2w and DWI sequences. This problem has been addressed recently by image reconstruction techniques employing DL networks that improve image quality (reduced image noise, increasing signal-to-noise ratio, increased sharpness) while drastically reducing scan time [8–10].

Currently, a rapid increase in MRI examinations for PCa is expected, and screening for PCa is heavily debated as promising effects, such as an increase in the diagnosis of PCa and detection of more localized tumor stages, were observed [11–14]. Current screening algorithms rely heavily on DRE and PSA levels with limited diagnostic accuracy and a high rate of overdiagnosis of non-csPCa, as well as a blind spot for PSA-negative csPCa [15–17]. Consequently, initial studies began to evaluate the benefit of PCa screening by MRI, showing improved diagnosis of csPCa without a gain in overdiagnosis of non-csPCa [18]. This effect was improved even further by the combination of MRI and PSA testing in a larger randomized controlled trial [19], although recent findings suggest that MRI screening may be of value independent of PSA, especially in the case of PSA-negative csPCa, even limiting overdiagnosis triggered by DRE and PSA testing [20].

This emphasizes the potential of MRI as an independent tool in screening for PCa and suggests that mpMRI may provide little diagnostic benefit given the additional time expense compared to abbreviated bp protocols.

We hypothesize that abbreviated bp PCa protocols can dampen the imminent spike in examinations while preserving diagnostic accuracy and improving patient comfort in a screening setting. A significant reduction in scan time could resolve the conflict of an increasing demand for screening examinations and limited capacity in clinical practice. Therefore, the aim of this study was to evaluate the diagnostic performance of an ultra-fast DL accelerated screening protocol compared to a standard multiparametric protocol.

## Materials and methods

### Study design

This mono-institutional prospective study was approved by the local ethics committee (22–1185) in compliance with the Declaration of Helsinki. It is listed in the German Clinical Trials Register (DRKS-ID: DRKS00029550). Written informed consent was obtained from all patients prior to examination.

### Study cohort

Consecutive biopsy-naïve patients that were referred to our department with a clinical suspicion of PCa (positive DRE, PSA-elevation above 4 ng/mL or positive PSA-dynamics of 0.3–0.7 ng/mL/year) between December 2022 and March 2023 were prospectively included in this study. Exclusion criteria were a) prior biopsy or treatment of the prostate, b) known PCa, c) incomplete MRI protocol, d) severe susceptibility or motion artefacts.

### MRI protocol

All examinations were performed in one 3-T scanner (MAGNETOM VIDA, Siemens Healthineers) in accordance with the PI-RADSv2.1 recommendations [3]. Patients were examined in a supine position without the use of an endorectal coil. All patients received (macrocyclic non-ionic) intravenous contrast agent with a bolus injection of 0.2 mL/kg body weight (Gadoteridol; 0.5 M, Bracco Imaging) at a flow rate of 2 mL/s, followed by a saline flush (25–30 mL). Scopolamine butylbromide was administered intravenously prior to the exam in order to reduce motion artefacts in the pelvis, if no contraindications were present. The standard mpMRI used in clinical routine consisted of a triplanar T2 TSE, axial DWI with calculated ADC maps and one extrapolated *b* value, axial T1 DIXON VIBE and DCE sequences (temporal resolution < 5 s). Additionally, research application sequences for DL axial T2w (DL-T2) and DWI (DL-DWI) were acquired prior to administration of CM. Refer to Table 1 for detailed acquisition parameters.

**Table 1:** Acquisition parameters

a) Comparison of deep learning accelerated sequences to standard sequences		
	Standard 2D T2 TSE	DL-T2
Orientation	axial	axial
Field of view [mm]	200 × 200	200 × 200
Matrix size [mm]	384 × 346	384 × 346
Number of slices	25	25
Slice thickness [mm]	3	3
Resolution [mm]	0.3 (i) × 0.3 (i)	0.3 (i) × 0.3 (i)
Interpolation	standard zerofilling	DL super resolution
PAT acceleration	GRAPPA	GRAPPA
Acceleration factor	3	4
Fat saturation technique	none	none
Averages	3	1
TR [ms]	7500	7440
TE [ms]	104	104
Phase Oversampling	100	182
Phase Resolution [%]	90	90
Flip Angle [°]	160	160
K-space to image reconstructions	standard GRAPPA reconstruction	DL reconstruction
Acquisition time [min:s]	03:54	01:26

	Standard DWI resolve	ZOOMit (DL-DWI)
Sequence type	rs-EPI	z-EPI
Orientation	axial	axial
Field of view [mm]	200 × 200	110 × 200
Matrix size [mm]	118 × 118	64 × 116
Number of slices	25	25
Slice thickness [mm]	3	3
Resolution [mm]	0.8 (i) × 0.8 (i)	0.9 (i) × 0.9 (i)
Interpolation	standard zerofilling	DL super resolution
Readout segments	5	1
Acceleration factor	2	1
Fat saturation technique	fatsat	fatsat
B-values [s/mm^2^]	50, 400, 1000, calculated 1400	50; 1000; calculated 1400
Diffusion directions	4	4
Averages	1/1/2	1/6
TR [ms]	4970	4300
TE [ms]	61	71
Phase oversampling	60	0
Phase resolution [%]	100	100
EPI factor	95	64
K-space to image reconstructions	standard GRAPPA reconstruction	DL reconstruction
Acquisition time [min:s]	05:34	02:02

b) Parameters of the additional sequences of the multiparametric protocol					
	T2 TSE	T2 TSE	T1 VIBE DIXON	T1 VIBE DIXON post CM	DCE (T1 VIBE grasp fs)
Orientation	coronal	sagittal	axial	axial	axial
Field of view [mm]	200 × 200	200 × 200	300 × 400	284 × 379	260
Matrix size [mm]	320 × 288	346 × 384	302 × 448	302 × 448	224
Number of slices	40	25	120	80	26
Slice thickness [mm]	3	3	2	3	3
Resolution [mm]	0.9 (i) × 0.9 (i)	0.3 (i) × 0.3 (i)	0.8 (i) × 0.8 (i)	0.8 (i) × 0.8 (i)	1.2 (i) × 1.2 (i)
K-space to image reconstructions	GRAPPA	GRAPPA	CAIPIRINHA	CAIPIRINHA	none
Acceleration factor	2	2	3	3	
Fat saturation technique	none	none	DIXON	DIXON	SPAIR
Averages	2	2	3	3	1
TR [ms]	7500	7440	5.4	5.4	4.1
TE [ms]	101	104	2.46/3.69	2.46/3.69	1.83
Phase oversampling	70	70	0	10	0
Phase resolution [%]	90	90	95	90	100
Flip angle [°]	160	160	9	9	12
Acquisition time [min:s]	02:24	03:08	03:35	02:22	04:48

*CAIPIRINHA* controlled aliasing in parallel imaging results in higher acceleration, *DL* deep learning, *EPI* echo-planar imaging, *fs* fat saturated; *GRAPPA* Generalized autocalibrating partially parallel acquisitions, *PAT* parallel acquisition technique, *rs* readout segmented multi-shot, *SPAIR* spectral attenuated inversion recovery, *TE* echo time, *TR* repetition time, *TSE* turbo spin echo, *z* zoomed single-shot

### DL reconstruction

For both DL-T2 as well as DL-DWI, the same approach of an unrolled reconstruction was used, following the idea of a variational network [21]. From raw k-space data and precalculated coil sensitivity maps that are provided as input, coil-combined images are formed in an iterative process that alternates between conventional, parallel imaging-based data consistency and DL-based image regularization. Two types of iterations are employed. First, several iterations focusing on data consistency without regularization were employed, followed by iterations, which additionally used a hierarchical down-up network as regularizer. For all iterations, trainable gradient step sizes and Nesterov-type extrapolation were used. T2 and DWI were reconstructed independently with respective implementations using slight variations of this general approach. Training of these two networks was performed offline in a supervised setting using volunteer data from different clinical 1.5-T and 3-T scanners (MAGNETOM, Siemens Healthineers, Erlangen, Germany). Ground truth target images were generated using all acquired k-space data, and the precalculated coil sensitivity maps input data were retrospectively undersampled. After offline training in PyTorch on a Nvidia Tesla V100-SXM2 GPU cluster, the networks were integrated in the scanner’s reconstruction framework.

For TSE, 18 iterations (7 without regularization, 11 with regularization) were executed in total, and training was performed using about 25,000 fully sampled images acquired with different contrasts in different body regions. For DWI, 17 iterations (6 without regularization, 11 with regularization) were executed in total, and training was performed on about 500,000 single-shot DWI images. No information between slices, *b*-values, or averages was shared. After DL reconstruction, DWI-specific steps, including ADC calculation, trace-weighting, averaging were performed analogous to the standard DWI processing.

### Image analysis

**Fig. 1:**
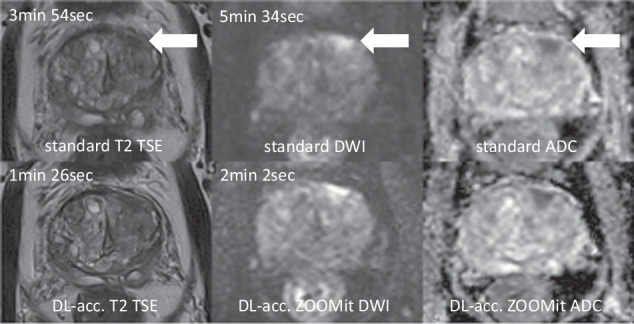
Imaging example of a suspicious lesion. Example of a PI-RADS 4 lesion in the anterolateral left transitional zone (white arrows depict the lesion; standard protocol in the upper row and accelerated screening protocol in the lower row). Required scan times for each sequence are provided in the upper left corners. Sequence types are specified on the bottom of each image. ADC, apparent diffusion coefficient; DL-acc., DL accelerated; TSE, turbo spin echo

Image assessment was performed with a commercially available PACS (DeepUnity, Daedalus). Relevant lesions were annotated by another radiologist (A.N.; 3 years of experience in mpMRI) prior to the reading sessions. Exams were evaluated independently by reader 1 (C.W.; 5 years of experience in mpMRI) and reader 2 (B.O.; 4 years of experience in mpMRI) in two reading sessions. In the first session, only the abbreviated protocol was available. Lesions were assigned a PI-RADS score according to the recommendation “assessment without adequate DCE” of the PI-RADS lexicon [3]. Afterwards, the overall image quality was rated using a five-point Likert scale [22] for a) overall quality, b) subjective image noise, c) clarity of the organ capsule, d) edge sharpness of anatomic boundaries of the prostate, e) microstructure in nodules, f) margin of nodules. Ratings ranged from 1–5 (1 = non-diagnostic; 2 = poor; 3 = moderate; 4 = good; 5 = excellent). In a second reading after two weeks, only the standard protocol was made available for evaluation in random order to reduce biases. Readers were blinded to the results of the first reading. This design was chosen to compare a drastically abbreviated screening protocol to the extensive multiparametric protocol and provoke potential differences in PI-RADS scoring, as there were no additional planes, anatomical T1-weighted images, or DCE sequences available in the first reading. Refer to Fig. 1 for a representative comparison of the standard and the abbreviated protocol.

### Statistical analysis

Data analysis was performed with the open-source software R Studio version 4.3.1 [23]. Sensitivity, specificity, positive predictive value (PPV), and negative predictive value (NPV) were calculated for the cut-off of PI-RADS ≥ 3 and PI-RADS ≥ 4, using mpMRI as the gold standard. Intra- and inter-observer agreements for PI-RADS scores and image quality were assessed using weighted Cohen’s k statistics to account for random agreement between readers and ordinal variables. A k < 0.21 was considered as poor agreement, 0.21–0.4 as fair agreement, 0.41–0.6 as moderate agreement, 0.61–0.8 as substantial agreement, and 0.81–1 as excellent agreement [24]. Image quality was compared using the Wilcoxon test for non-normal distributed data [25]. *p* values ≤ 0.05 were considered as statistically significant.

## Results

### Patients

**Fig. 2:**
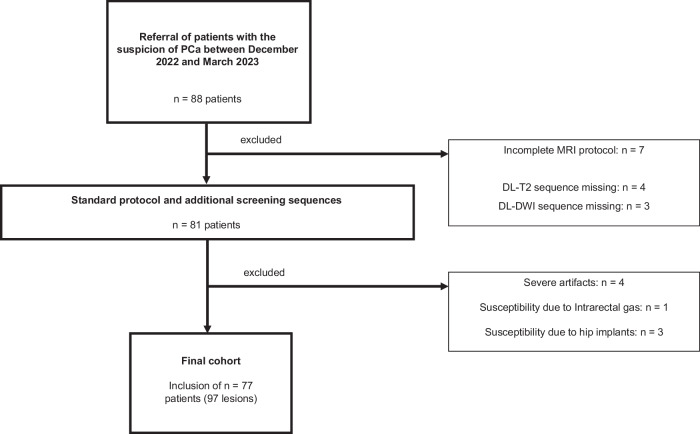
Flow chart of the patient selection process

Eight-eight patients were prospectively enrolled in this study. Seven patients were excluded due to an incomplete MRI protocol, and four patients due to severe artefacts and nondiagnostic imaging. The final cohort consisted of 77 patients with 97 lesions. Mean age was 66 years (range: 50–84; SD: 7.7), median PSA levels were 7.0 ng/mL (IQR: 5.04–9.95). For detailed information, refer to Fig. 2.

### Scan time

By reducing the full multiparametric protocol to a minimum of two DL-accelerated sequences, we shortened the total scan time from 25 min 45 sec to 3 min 28 sec (14%). The standard axial T2 TSE took 3 min 54 sec compared to the DL-T2 with 1 min 26 sec (37%). Standard DWI was acquired in 5 min 34 sec compared to 2 min 2 sec (37%) for the ZOOMit sequence.

### Image analysis

**Fig. 3:**
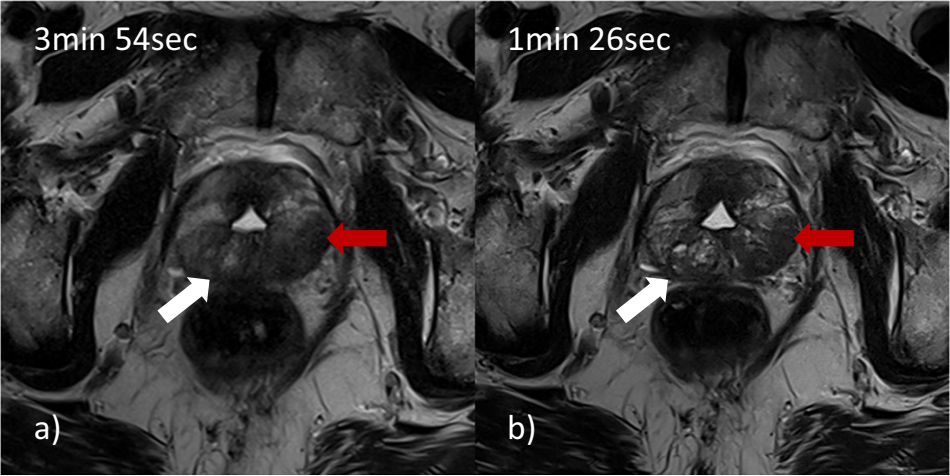
Comparison of standard T2 and DL-T2. **a** Standard axial T2w image of the pelvis: Note blurring around the posterior prostatic capsule (including the suspicious lesion) and the rectal cavity due to motion artefacts; **b** Axial DL-T2 image of the same region: Note superior clarity of the organ capsule, better edge sharpness of the lesion and clear visibility of microstructure in the hyperplastic nodule on the right-hand side. Red arrow: lesion suspicious for PCa; white arrow: hyperplastic nodule; required scan times for each sequence are provided in the upper left corners

All included images were read as sufficiently diagnostic regarding quality. Distribution of PI-RADSv2.1 scores for R1 and R2 were as follows: 2 lesions vs. 3 lesions PI-RADS 1, 33 lesions vs. 33 lesions PI-RADS 2, 20 lesions vs. 21 lesions PI-RADS 3, 30 lesions vs. 26 lesions PI-RADS 4, 12 lesions vs. 14 lesions PI-RADS 5 for the abbreviated protocol and 2 lesions vs. 3 lesions PI-RADS 1, 34 lesions vs. 39 lesions PI-RADS 2, 19 lesions vs. 11 lesions PI-RADS 3, 30 lesions vs. 31 lesions PI-RADS 4, 12 lesions vs. 13 lesions PI-RADS 5 for the standard protocol implemented in clinical routine. The sensitivity and specificity of the abbreviated protocol (Cut-off PI-RADS ≥ 3) were 100%/97% for R1 and 98%/83% for R2, respectively. Sensitivity and specificity (cut-off PI-RADS ≥ 4) were 100%/100% for R1 and 89%/98% for R2. For detailed information, refer to Tables 2 and 3. Mean image quality was 4.74 for R1 and 4.57 for R2 for the abbreviated protocol vs. 3.96 and 4.35 for the standard protocol (*p* < 0.05). Refer to Fig. 3 and Table 4 for detailed information.

**Table 2:** Diagnostic performance

	Reader 1				Reader 2			
	Cut-off PI-RADS ≥ 3	95%CI	Cut-off PI-RADS ≥ 4	95%CI	Cut-off PI-RADS ≥ 3	95%CI	Cut-off PI-RADS ≥ 4	95%CI
Sensitivity	1.00	(0.94, 1.00)	1.00	(0.92, 1.00)	0.98	(0.90, 1.00)	0.89	(0.75, 0.96)
Specificity	0.97	(0.85, 1.00)	1.00	(0.94, 1.00)	0.83	(0.69, 0.93)	0.98	(0.90, 1.00)
PPV	0.98	(0.91, 1.00)	1.00	(0.92, 1.00)	0.89	(0.78, 0.95)	0.98	(0.87, 1.00)
NPV	1.00	(0.90, 1.00)	1.00	(0.94, 1.00)	0.97	(0.85, 1.00)	0.91	(0.81, 0.97)

Comparison of diagnostic performance between reader 1 and reader 2 for the given thresholds with the multiparametric protocol as reference standard. *CI* confidence interval, *PI-RADS* Prostate Imaging Reporting and Data System, *PPV* positive predictive value, *NPV* negative predictive value

**Table 3:** Distribution of assigned PI-RADS scores

PI-RADS score	Abbreviated protocol		Conventional protocol	
	Reader 1	Reader 2	Reader 1	Reader 2
1	2	3	2	3
2	33	33	34	39
3	20	21	19	11
4	30	26	30	31
5	12	14	12	13

*PI-RADS* Prostate Imaging Reporting and Data System

**Table 4:** Distribution of assigned Likert scores

Likert score	Abbreviated protocol		Conventional protocol	
	Reader 1	Reader 2	Reader 1	Reader 2
1	0	0	0	0
2	1	1	1	0
3	2	5	14	9
4	13	20	49	32
5	61	51	13	36

1 = non-diagnostic, 2 = poor, 3 = moderate, 4 = good, 5 = excellent

### Inter-reader agreement

Inter-reader agreement was moderate (*κ*: 0.55; 95CI: 0.43–0.68) for the screening protocol and substantial (*κ*: 0.61; 95CI: 0.49–0.74) for the mp protocol. Intra-reader agreement was excellent (*κ*: 0.98; 95CI: 0.96–1) for R1 and substantial (*κ*: 0.79; 95CI: 0.69–0.89) for R2.

## Discussion

We evaluated the diagnostic performance and quality of an ultra-fast DL accelerated screening protocol in biopsy-naïve men compared to a standard mp protocol as used in the current clinical routine. The abbreviated bp protocol was considered diagnostic in all patients and showed no significant difference in diagnostic performance compared to the mp protocol. Furthermore, the image quality of the abbreviated protocol was rated as better by both readers. Those results are in line with several studies which evaluated DL-sequences for MRI of the prostate, although neither evaluated an entirely DL-accelerated screening protocol but merely individual DL-accelerated sequences or DL-accelerated sequences as part of a conventional protocol [26–29].

Considering the standard mp protocol as the reference standard, both sensitivity and specificity were excellent for R1. These results are also reflected by Kuhl et al who found similar diagnostic accuracy for abbreviated bpMRI and mpMRI. Only one additional csPCa was detected in mpMRI compared to bpMRI as opposed to 11 additional false-positive diagnoses [30]. Van der Leest et al found that an abbreviated biparametric protocol resulted in a surplus of 2% in biopsies and 1% overdetection of low-grade PCa compared to the standard protocol with similar sensitivity (95%) and a slightly lower specificity (65% compared to 69%) in 626 patients [31].

Employing a cut-off of PI-RADS ≥ 3, R2 achieved an equally high sensitivity at the cost of specificity (98%/83%). An adverse effect was observed regarding a threshold of PI-RADS ≥ 4, lacking sensitivity instead (89%/98%). This effect may be due to slightly less experience in reporting prostate MRI [32]. Coherently, this is reflected in inter-reader agreement, showing only substantial agreement between readers at most (*κ*: 0.61), whereas intra-reader agreement was substantial to excellent. This implies that different PI-RADS scores between readers most likely emerge from varying interpretations of lesion characteristics rather than from differences in image quality. Additionally, we observed an unequal distribution of PI-RADS scores in both the abbreviated and the mp protocol for both readers (refer to Table 3). In particular, barely any PI-RADS 1 lesions and only a few PI-RADS 5 lesions were included. This limits generalizability for PI-RADS 1 lesions but implies robust diagnostic performance for PI-RADS 2–4 lesions. These lesions tend to be most debatable in clinical practice due to the possibility of an upgrade in case of positive DCE or higher DWI categories. Heterogeneity in the distribution of PI-RADS scores between readers was higher in the mp protocol. This finding was to be expected as the mp protocol offers more options to inspect the lesion in its three-dimensional shape and to upgrade scores via DCE.

We consider that enhanced image quality is mainly due to the reconstruction of the DL-algorithm, but also critically related to the reduced acquisition time, resulting in less motion artefacts [33]. We used the resolve DWI as a reference, as it serves as our routine sequence for prostate exams. To find a shorter alternative sequence a single shot EPI technique was chosen, which is more time efficient compared to the segmented readout resolve acquisition. By integrating a DL reconstruction in the EPI sequence scan time could be even further cut down. Besides the reduction of motion artefacts another potential benefit of the reduction of scan time is an increase in patient comfort and compliance, especially valuable in an elder patient collective as laying still becomes an increasing problem during the examination period [34, 35]. Abbreviated protocols are especially suitable for specific indications that do not require extensive three-dimensional information or contrast enhancement, e.g., screening for PCa. This offers higher MRI throughput with increased expediency at a potentially lower cost per examination, given the indication is chosen correctly [36]. However, single-planar protocols may not suffice for a more detailed anatomical evaluation of the tumor, potential extracapsular growth, or local staging [37, 38]. Adequate resolution in a single axial plane already resolved issues of volume measuring or co-registration of the gland for targeted biopsy in our institution, although abbreviated protocols have yet to be evaluated thoroughly for tumor segmentation prior to local therapy, follow-up examinations and assessment of local tumor recurrence [39]. Another disadvantage of providing more than one standard protocol or even numerous variants is a potential increase in the complexity of protocol selection within the clinical workflow, possibly resulting in inadequate examinations [40]. Furthermore, indefinite results of abbreviated protocols and recall of patients for additional examinations may limit their acceptance. Consequently, in order to exploit the advantages of shorter scan times, clinical workflows, as well as indications for examinations need to be further refined [41].

Although carried out prospectively, the study was conducted in a single centre setting with a relatively small cohort. Furthermore, seven patients were excluded due to incomplete scans and four patients due to severe artefacts. While effects from incomplete scans represent a random error and artefacts affected both the abbreviated and the multiparametric protocol and effects, this might slightly distort the initial cohort and limit generalizability further. Despite the fact that these initial results suggest a benefit in image quality and imaging efficiency at an adequate diagnostic performance, external validation is required, ideally in a multi-center setting.

As we compared slightly different sequence types of DWI (readout-segmented DWI, in this case, RESOLVE as currently implemented standard vs. ZOOMit single shot EPI), direct comparability might be limited. In the ZOOMit sequence, regions outside of the field of view do not contribute to the gathered MR signal, therefore preventing infolding of signals outside of the field of view. Consequently, motion artefacts are reduced to a minimum [42]. A recent study by Klingebiel et al suggests sufficient comparability as they showed that zoomed sequences outperformed conventional multi-shot readout sequences regarding objective image quality [43].

Omitting DCE sequences in the abbreviated protocol might hinder the upgrade of PI-RADS 3 scores to PI-RADS 4 in the peripheral zone compared to the multiparametric protocol. However, recent evidence suggests limited benefit of upgrading those lesions as they seem to yield significantly less csPCa compared to PI-RADS 4 lesions that were not upgraded (14.5% csPCa in PI-RADS 3 with positive DCE and 53.3% csPCa in PI-RADS 4 lesions) and decrease detection rates for csPCa [4]. The authors suggest reconsidering the current scoring categorization and propose to set the indication for biopsy according to the PSAD for PI-RADS 3 lesions with positive and negative DCE alike. These findings generally question the implementation of DCE imaging in PI-RADS scoring but even more so justify the omission in a screening setting as a tradeoff for efficiency.

Furthermore, we compared the abbreviated protocol to the current gold standard for imaging of PCa (mpMRI) without histopathological confirmation, so we could only evaluate diagnostic performance but not diagnostic accuracy. Although we found no significant difference between the abbreviated and the standard protocol and similar results can be expected, validation studies are desirable. Currently, there is a general lack of evidence in the comparison of abbreviated and standard protocols, requiring further research. Moreover, additional investigation of implementation and practicability in clinical routine and cost-effectiveness will be necessary.

In conclusion, an abbreviated screening protocol for prostate cancer in biopsy-naïve men proved similar diagnostic performance and better imaging quality compared to the conventional multiparametric protocol, requiring less than 15% of scan time. This results in a major improvement in imaging efficacy, enabling the implementation of screening protocols in clinical routine.

## Abbreviations

ADC: Apparent diffusion coefficient
Bp: Biparametric
CM: Contrast medium
DCE: Dynamic contrast enhanced
DL: Deep learning
DRE: Digital rectal examination
DWI: Diffusion weighted imaging
Mp: Multiparametric
MRI: Magnet resonance imaging
PCa: Prostate cancer
PI-RADS: Prostate Imaging Reporting and Data System
PSA: Prostate specific antigen
PSAD: Prostate specific antigen density
R: Reader
T2w: T2-weighted
TSE: Turbo Spin Echo
